# Exploring the Reliability and Validity of the Huntington’s Disease Quality of Life Battery for Carers (HDQoL-C) within A Polish Population

**DOI:** 10.3390/ijerph16132323

**Published:** 2019-06-30

**Authors:** Agnieszka Bartoszek, Aimee Aubeeluck, Edward Stupple, Adrian Bartoszek, Katarzyna Kocka, Barbara Ślusarska

**Affiliations:** 1 Department of Family Medicine and Community Nursing, Medical University of Lublin, Staszica 6 Str., 20-081 Lublin, Poland; 2 School of Health Science, Royal Derby Hospital, University of Nottingham, Nottingham NG7 2RD, UK; 3 Division of Psychology, University of Derby, Derby DE22 1GB, UK; 4 Department of Biochemistry, Faculty of Medicine, Medical University of Lodz, 90-419 Lodz, Poland

**Keywords:** Huntington’s disease, quality of life, family caregiving, reliability and validity, factor analysis, Poland

## Abstract

Huntington’s disease (HD) is a rare genetic neurodegenerative disorder that causes motor disorders, neuropsychiatric symptoms and a progressing deterioration of cognitive functions. Complex issues resulting from the hereditary nature of HD, the complexity of symptoms and the concealed onset of the disease have a great impact on the quality of life of family carers. The caregivers are called the “forgotten people” in HD, especially with relation to genetic counseling. This study aims to explore the reliability and validity of the Huntington’s Disease Quality of Life Battery for carers (HDQoL-C) within a Polish population. A total of 90 carers recruited from the Enroll-HD study in Polish research centers of the European Huntington’s Disease Network completed a polish translation of the HDQoL-C. Data were subjected to Principle Components Analysis (PCA) and reliability measures. The Polish version of the shortened versions of the HDQoL-C is similarly valid compared to the original English version and suitable for use within this population. The HDQoL-C has previously demonstrated a wide range of benefits for practitioners in capturing and understanding carer experience and these benefits can now be extended to Polish speaking populations.

## 1. Introduction

Huntington’s disease (HD) is a rare genetic neurodegenerative disorder caused by the mutation of the IT15 gene, which codes the huntingtin protein located on the short arm of chromosome 4 [1]. The clinical presentation of the disease includes motor disorders, neuropsychiatric symptoms and a progressing deterioration of cognitive functions. The motor symptoms consist of involuntary choreatic movements and impaired saccadic eye movements. The symptoms that appear at the subsequent stages of the disease are dystonia, dysarthria, dysphagia, rigidity and bradykinesia, leading to death in 15–20 years [2,3]. The most frequent causes of death are aspiration pneumonia, injuries resulting from falls and suicides, which are recorded twice as often as in the total population [4]. In the juvenile form that usually presents with mental degradation, motor disorders occur later and the course of the disease is more acute as death usually occurs 8–10 years from the onset of the first symptoms. The frequency of neuropsychiatric symptoms in HD is 33–76%, of which the most frequent are: depression, anxiety disorders, irritability, apathy, obsessive-compulsive disorders and psychotic symptoms [5,6]. Cognitive disorders include memory deterioration, slowed thinking processes, disorders of executive and visual and spatial functions, problems with organization, planning and multitasking, difficulties with decision-making and with dealing with new situations [7].

Despite the fact that diagnosing HD is traditionally based on motor symptoms, the highest impact on patients and carers occurs due to the patient’s mental state and behavior changes [8,9] as well as cognitive disorders, which may appear as early as 12–15 years before the diagnosis and delay an accurate diagnosis of HD [10,11].

Due to its autosomal dominant type of inheritance, HD is passed over from generation to generation and has damaging effects both on the patients and their carers/families, who usually become responsible for caring for their family member with HD [12]. The disease’s trajectory in the family may last up to 30 years or even more [13]. The literature indicates that for each person with HD, up to 20 family members bear various consequences of the disease [14,15]. These family members include persons at risk of the disease as well as people who experience tension due to having to inform their children and other relatives of the risk of becoming ill. They blame themselves for passing on the HD gene and for the fact that every child can contract the disease, which may have a different form and varying clinical symptoms. They are struggling with a difficult decision related to children planning. They are also aware that in the future, they may need to look after a few generations (parents, spouse, siblings, children), possibly several persons at the same time [12,15,16,17]. HD is a paradigmatic example of a family disease [18].

Complex issues resulting from the hereditary nature of HD, the complexity of symptoms and the concealed onset of the disease [19] have a great impact on the quality of life of family carers [20], who are aware that the disease might develop in other family members. Carers blame themselves for passing the disease on to the next generation [13,21,22,23].

For each child in a family with a parent having HD, the risk of inheriting the gene is 50% [12]. Diagnostic and preclinical trials can clearly confirm the genetic status of a person [1]. Carers fear that they might be forced to provide care for several generations and for a few people at the same time, with such a situation possibly lasting for years [24].

There are few studies that directly investigated the impact of HD on the quality of life (QoL) of family carers [25]. The existing studies demonstrated that the quality of life of carers is seriously compromised due to this burden [26]. These capture a unique sense of long-term isolation and frustration [14]. These feelings are connected to the performed role and conflicting roles. On the one hand, they need to care for their partner and on the other, carers need to protect their children from unexpected behavior, irritability or aggression of the HD patients [16]. Insufficient resources to perform these duties of care are also highlighted as being important [17,27,28]. Carers experience substantial changes in communication with HD patients [29] and their ability to perform care and parental functions deteriorates as the disease progresses [30,31] and as their physical and mental health declines [22].

The quality of life of carers is considerably reduced due to the lack of access to specialized medical care, while medical personnel are often unaware of the immense impact of HD on the family and unprepared to solve specific problems resulting from the complex changes in HD families [32]. Feeling abandoned by the system, carers describe their experiences with medical professionals as “a lone journey” [28,33]. Family carers were described as “the forgotten” both in the families with HD [34], and in the genetic counselling system [35].

As HD is currently incurable and there are no effective treatment methods, the concept of the quality of life is particularly important due to the unique burden placed on carers. Using specific questionnaires to evaluate the QoL of carers makes it possible for physicians and researchers to assess the emotional and physical functioning and lifestyle from the carer’s perspective, which does not provide an objective interpretation [25]. It offers a possibility to assess changes in the QoL of carers over time. In particular, despite many promising treatment options, the disease is currently incurable, and one thing is certain: the path towards the solution is long [36].

The existing literature highlights the burden that family carers may face in supporting a loved one living with HD but as yet, there is no validated quality of life measure to assess the impact of caring on the QoL of family members who are caregivers for a loved one with HD in Poland. In Poland, there is a lack of studies focusing on the impact of HD on the quality of life of family carers.

According to our knowledge, this is the first study that focuses on such a large group of Polish family carers with the use of a specific scale for evaluating the quality of life of carers in HD. Thus far, only two reports referring to Polish carers of HD patients have been produced [27,37].

The aim of this study was therefore to validate a translated version of the Huntington’s Disease Quality of Life battery for carers (HDQoL-C) [20] for use in Poland.

## 2. Materials and Methods

### 2.1. Instrumentation

The HDQoL-C is a multidimensional, disease-specific and subjective health related quality of life tool that incorporates the individual’s physical health, psychological state, level of independence, social relationships and personal beliefs. The scale was developed as an outcome measure and can also be used to assess subjective QoL in family carers of people with HD. Cronbach’s alpha scores for the three components of the original HDQoL-C scale demonstrate good internal consistency as they were 0.801 (Practical aspects of caregiving); 0.844 (Satisfaction with life) and 0.885 (Feelings about living with HD), with test-retest reliability for the same components of 0.86, 0.90 and 0.92, respectively [20]. The scale demonstrates good congruent validity, good face validity and robust content validity. It has also been translated and validated with success into French and Italian [25]. The HDQoL-C is being used in ongoing Euro-Huntington’s Disease Burden studies to measure the impact of Huntington’s Disease in several European countries and in the USA. To develop the Polish version of the HDQoL-C, forward-backwards translation methods were applied to the original English version of the scale. The translation did not show any errors in translation.

### 2.2. Participant and Procedure

Participants were recruited among carers taking part in the Enroll-HD study in Polish research centers of the European Huntington’s Disease Network. Contact with carers was also established via the Polish Huntington’s Disease Association during the annual conference in Warsaw, Poland that is devoted to HD, with the carers involved in adding new members to the surveyed group, and through the “forum―HD zamki” website. The selection criteria of carers for the study was an age ≥ 18 years, the presence of a patient living at home and written consent to participate in the study. All carers who took part in the study had a loved one under their care who had tested positive for HD.

From 100 carers contacted directly, through post or electronic mail, 90 responded to the invitation to take part in the study, which was carried out from June 2015 to December 2016. A total of 18 out of 20 carers answered a retest questionnaire after a 2-week interval to gather data on test–retest reliability. Furthermore, as HD is a rare disease and there are no detailed data indicating the prevalence of disease in Poland, the sample size is likely to be adequate for the aims of this study. Ethical approval to conduct this study was granted by the Bioethics Committee of the Medical University of Lublin, Poland, (Protocol number KE-0254/134/2015). Written informed consent was obtained from each participant.

### 2.3. Data Analysis

Principal components analysis (PCA) was used to explore the inter-relationship between the variables on the HDQoL-C. Bartlett’s test of sphericity and the Kaiser–Meyer–Oklin (KMO) measure of sampling adequacy were also used to assess the suitability of the data for PCA.

Cronbach’s α coefficient was used to assess the internal consistency of the scale. A reliability threshold level was considered acceptable when α was greater than 0.70. Reproducibility assesses if an instrument produces the same results on repeated administrations when respondents have not changed. The reliability coefficient was computed by correlating instrument scores for the two administrations.

## 3. Results

### 3.1. Respondent Characteristics

The sample was comprised of 90 carers. A total of 57.8% of carers were the main carers of a HD patient. The carer ages ranged between 20 and 80 years, with a mean of 48.78 years. Most carers were women (68%) and were not carers before (81%). A total of 57.8% carers were married and 41.1% had a child with a risk of disease. Mean length of caring for an HD affected family member was 7.81 years. Table 1 presents the characteristics carers in family with HD.

### 3.2. Principle Components Analysis and Reliability

Consistent with the English language version, separate PCAs were conducted. Section 1 of the questionnaire is comprised of demographic information and thus was not included in the analysis. For Section 2, the Kaiser-Meyer Olkin measure of sampling adequacy showed that the sample was factorable (KMO = 0.714). Bartlett’s Test of Sphericity was highly significant (χ^2^ = 210.4, df = 36, *p* < 0.001), and low off-diagonal values in the anti-image correlation matrix demonstrated that the data were suitable for factor analysis [38]. The analysis revealed two factors. The cross loadings displayed for item QoL2_2 in Factor 1 may indicate cause for concern as it also negatively loaded on to factor 2 at −0.334. Table 2 outlines the Pattern Matrix of rotated factor loadings for section 2.

Internal consistency was analyzed using Cronbach’s Alpha. The items in Factor 1 demonstrated high reliability (Cronbach’s α = 0.81). Factor 2 demonstrated moderate reliability (Cronbach’s α = 0.58), which increased to α = 0.64 if item QoL2_9 was deleted.

The PCA for Section 3 showed a Kaiser-Meyer Olkin measure of sampling adequacy, indicating that the sample was factorable (KMO = 0.858). Bartlett’s Test of Sphericity was highly significant (χ^2^ = 354.3, df = 28, *p* < 0.001), and low off-diagonal values in the anti-image correlation matrix demonstrated that the data were suitable for factor analysis. The analysis produced a two-factor solution although the second factor only featured a single item (Qol3_7). Table 3 outlines the Pattern Matrix of rotated factor loadings for section 3.

PCA for section 4 indicated a Kaiser-Meyer Olkin measure of sampling adequacy such that the sample was factorable (KMO = 0.795). Bartlett’s Test of Sphericity was highly significant (χ^2^ = 569.2, df = 136, *p* < 0.001), and low off-diagonal values in the anti-image correlation matrix demonstrated that the data were suitable for factor analysis [39]. A four-factor solution was indicated although the items in the fourth factor were problematic with high cross loadings. Table 4 outlines the Pattern Matrix of rotated factor loadings for section 4.

Internal consistency was again analyzed using Cronbach’s Alpha. The items in Factor 1 demonstrated high reliability (Cronbach’s α = 0.85). Factor 2 demonstrated good reliability (Cronbach’s α = 0.70), Factor 3 had moderate reliability (Cronbach’s α = 0.51). Factor 4 was moderately reliable (Cronbach’s α = 0.56). However, if item QoL4_2 was excluded, the reliability of this sub-scale increased considerably (Cronbach’s α = 0.78).

### 3.3. Test-Retest

A total of 18 caregivers filled the questionnaire again after two weeks. Coefficients are presented in Table 5. All components present high statistically significant correlation (*p* < 0.001) with satisfying coefficients of determination (r^2^ > 0.6).

## 4. Discussion

By measuring QoL in this Polish population, we were able to build on our understanding of the issues surrounding caregiving in HD in order to establish ways of improving QoL for this carer group. The translation of the HDQoL-C into an additional language means that the scale can be used even more widely, allowing for further comparisons across Europe. The need to translate and adapt QoL instruments for use in languages other than the source language (usually English) has increased with the internationalization of clinical trial programs and cross-cultural research. For example, the ISPOR Task Force [38] have noted the importance of evidencing similarities in measurement properties between all versions of the same tool to pool analysis and facilitate comparability between countries. The HDQoL-C has previously been translated into French and Italian. The tested components showed a high degree of reliability (Cronbach’s alpha coefficients were found to be 0.88 for the ‘‘Satisfaction with life’’ component and 0.8 for the ‘‘feelings about living with HD’’ component). No differential item functioning between France and Italy was detected according to the Zumbo criteria [25]. The Polish version of the shortened versions of the HDQoL-C is similarly valid compared to the original English version and is a tool with satisfactory psychometric properties that may be used for the group of HD carers. Cronbach’s alpha scores for the three components of the original HDQoL-C scale demonstrate good internal consistency as they were 0.801 (Practical aspects of caregiving), 0.844 (Satisfaction with life) and 0.885 (Feelings about living with HD), with test–retest reliability for the same components of 0.86, 0.90 and 0.92, respectively [20].

The addition of this Polish translation has the potential to further our understanding of any cross-cultural differences in the resources, policies and practices that may influence the QoL HD family carers at a global level.

The scale instrument showed satisfactory face validity with little missing data (1.1%). Cronbach’s alpha coefficients demonstrate moderate to good reliability. There were two items, “How often do you sleep well?” (section 2, question 9) and “I feel financially disadvantaged” (section 4, question), that increased the reliability of the subsection they sit within if they were removed. The question “How often do you sleep well?” has consistently reduced factor reliability even in the original version and was kept in due to the emphasis that carers have placed on it despite not functioning well in the factor analysis. It may be possible that this question needs refinement in terms of wording to improve reliability or that the subjectivity of this item is difficult to articulate. With regards to the statement “I feel financially disadvantaged”, it may be that this statement does not translate cross-culturally or is not relevant for a Polish population. It may also be the case that the Polish carers were less focused on financial disadvantages than on the heritable or familiar elements of their experience of caring for someone with HD.

It should also be emphasized that the contact with carers during the collection of data for the study was of a unique nature and had a therapeutic effect on them. They expressed that they were pleased that their roles were recognized as most measures focus on HD patients. This is in line with evidence from reference [20] who also observed a cathartic benefit of engaging with family carers, many of whom described completing the questionnaire as an intervention itself. Due to the fact that carers consider themselves forgotten patients in the healthcare system, there is a strong need to support caregivers in their role in order to find ways to improve their QoL.

The recommended solutions to help carers should include: health assessment, assistance in planning and providing care, providing caregivers with information about HD, ways of dealing with the disease, and the availability of psychological support and practical assistance. At the same time, it should be emphasized that such aid is required from the moment of diagnosis throughout the duration of the disease [13]. In addition to changes in legal regulations, intensive education is necessary regarding the organization of rehabilitation as well as logopedic and dietetic practice. There is also a need to spread a protective umbrella over the families of patients and to organize support groups for families with HD [40]. It should be emphasized that carers who receive support from their community perceive their caring tasks more positively and have a greater sense of control [18]. A lack of items bias in English and Polish translations confirms the scale’s multi-lingual, multi-cultural consistency and indicates that the scale is easily applicable in other languages. The Polish version of the HDQoL-C demonstrated good internal consistency and congruent validity. Further validation, such as test–retest validity and sensitivity to changes, would enhance this validation process.

## 5. Study Limitations

Although this is the first study in Poland with the use of the HD-specific quality of life questionnaire for family carers, it has some limitations. The group of carers was quite small (90 family carers, only 10 did not respond to the invitation to study) due to the rare occurrence of HD. It should be emphasized that access to individual caregivers is difficult and it is only possible during a conference that is organized annually by the Polish Huntington’s Disease Association (only those who benefit from such support) and an online forum during which there is no possibility of direct conversation. However, the most important thing is that during conversations at the conference and in subsequent telephone conversations, the carers emphasized that just filling out the questionnaire and contacting researchers was a positive experience for them as they had the opportunity to verbalize their feelings and difficulties related to the role of a caregiver. This is confirmed by the comments at the end of the questionnaire, in which the caregivers thanked for noticing them and understanding. At the same time, they emphasized that they do not have the closest people with whom they could talk, because the family avoids topics about HD. Of the entire study group, only 7 people did not leave contact, which also indicates their needs. The group of 90 respondents was also created thanks to carers who willingly provided information to other caregivers.

## 6. Practice Implications

Interest in the QoL of carers is crucial due to the practical, socioeconomic aspects. In the situation when care-related costs are borne mainly by carers and not the State budget, incapacities of carers generate unplanned expenses for medical and social assistance [41]. Due to the fact that carers feel like a forgotten group in HD families, it is necessary to promote quality of life in primary healthcare. The time of caring for a patient with HD is much longer than in other neurodegenerative diseases and the need for environmental care is also longer. It is recommended that the quality of life of HD caregivers in primary healthcare should be further studied in order to implement appropriate support procedures at various stages of the disease, especially considering that carers can also get ill and pass defective genes to their children.

## 7. Research Recommendations

This study is part of a wider project on family carers in HD in Poland. Further research, with quantitative and qualitative approach, can identify other areas of quality of life for HD families. Moreover, due to the multitude of symptoms (multifaceted disease), conducting interdisciplinary research would provide a better understanding of the needs of family carers and this would translate into providing them with practical support. Therefore, further research based on mixed methodology and conducted within multidisciplinary teams is recommended.

## 8. Conclusions

A lack of item bias in English and Polish translations confirms the scale’s multi-lingual, multi-cultural consistency and indicates that the scale is easily applicable in other languages. The Polish version of the HDQoL-C demonstrated good internal consistency and congruent validity. Further validation, such as sensitivity to changes, would enhance this validation process. The HDQoL-C has demonstrated a wide range of benefits for practitioners in capturing and understanding carer experience and these benefits can now confidently be extended to Polish speaking populations.

## Figures and Tables

**Table 1:** Characteristic of the researched group family carers (*n* = 90).

Characteristics of Family Carers	*n*	%
Women	61	68
Age (years)	48.78 (±15.21)	
Main carer	52	57.8
Marital status		
Married	52	57.8
Single	17	18.9
Widowed	13	14,4
Partnership	5	5.6
Divorced	3	3.3
Family situation		
Number of years since HD knowledge in family	11.76 (±10.4)	
Have children at risk	37	41.1
Relation with HD patient		
Husband/wife	31	34.4
Parent	26	28.9
Child	14	15.6
Other	10	11
Sibling	8	8.9
Partner	1	1.2
Carer background		
Carer has previously cared any other HD affected person	17	19
Duration of caring (in years)	7.81 (±8.48)	

**Table 2:** Pattern Matrix of rotated factor loadings for section 2.

Item	Content	Factor	
		1	2
Qol2_3	How often do you have access to professionals that have specialized knowledge of HD and understand its implications?	0.827	–0.072
Qol2_4	How much support are you given by health care professionals?	0.788	–0.030
Qol2_6	How often do you have access to appropriate care facilities?	0.766	0.004
Qol2_7	How often do you receive any practical support you need?	0.754	0.036
Qol2_2	How often do you receive appropriate help from social services?	0.610	–0.334
Qol2_1	How often are you restricted by the need to maintain a regimented daily routine?	0.039	0.816
Qol2_8	How often do you experience a conflict of interest between what you want and what your HD affected relative wants?	0.067	0.741
Qol2_5	How often does the inherited nature of HD further complicate your caring role?	–0.117	0.667
Qol2_9	How often do you sleep well?	–0.087	0.337

**Table 3:** Pattern Matrix of rotated factor loadings for section 3.

Item	Content	Factor	
		1	2
Qol3_2	How satisfied are you with what you achieve in life?	0.860	–0.128
Qol3_8	How satisfied are you with your overall quality of life?	0.825	0.246
Qol3_4	How satisfied are you with how safe you feel?	0.811	0.223
Qol3_1	How satisfied are you with your health?	0.792	0.000
Qol3_6	How satisfied are you with your own happiness?	0.763	0.329
Qol3_5	How satisfied are you with feeling a part of your community?	0.656	0.373
Qol3_3	How satisfied are you with your close relationships with family or friends?	0.566	0.464
Qol3_7	How satisfied are you with the treatment that your HD affected relative receives?	0.019	0.926

The items in Factor 1 demonstrated high reliability (Cronbach’s α= 0.90).

**Table 4:** Pattern Matrix of rotated factor loadings for section 4.

Item	Content	Factor			
		1	2	3	4
Qol4_7	I feel sad or depressed	0.775	0.052	0.310	0.097
Qol4_17	I feel like I don’t know who I am anymore	0.765	0.087	–0.017	0.006
Qol4_8	I feel stressed	0.749	0.051	0.143	0.129
Qol4_5	I feel exhausted	0.729	0.126	0.186	–0.161
Qol4_16	I feel that I have had a “duty of care” forced on me	0.711	–0.166	–0.269	0.213
Qol4_10	I feel my own needs are not important to others	0.645	–0.063	0.400	–0.157
Qol4_3	I feel isolated	0.592	0.213	0.282	–0.154
Qol4_4	I feel there is hope for the future	0.036	0.797	0.287	0.004
Qol4_11	I feel comforted by the belief that one day there will be a cure for HD	0.024	0.773	–0.155	0.160
Qol4_13	I feel comforted by my beliefs	0.060	0.674	0.084	0.253
Qol4_9	I feel worried about the genetic consequences of HD	0.038	–0.024	0.793	0.131
Qol4_1	I feel guilty	0.220	0.080	0.590	–0.006
Qol4_6	I feel supported	0.176	0.386	0.474	0.125
Qol4_12	I feel that HD brought something positive to my life	–0.029	0.334	0.076	0.701
Qol4_15	I feel that HD has made me a stronger person	0.166	0.404	0.246	0.673
Qol4_2	I feel financially disadvantaged	0.424	0.179	0.170	–0.583
Qol4_14	I feel that I can cope	0.282	0.322	0.467	0.546

**Table 5:** Test–retest correlations for sub-scales.

Sub-scales	II	III	IV
II	0.82	-	-
III	-	0.92	-
IV	-	-	0.83

## Data Availability

The HDQoL-C is the property of Dr Aimee Aubeeluck, CPsychol, FHEA. It has been developed for use by family members, researchers and clinicians and can be used and adapted freely for the benefit of improving the quality of life of families living with Huntington’s Disease. To use the scale, contact is required: aimee.aubeeluck@nottingham.ac.uk and cite in any subsequent write up.
